# Supplementary material for: Phytohormone cytokinin guides microtubule dynamics during cell progression from proliferative to differentiated stage
Source: EMBO J. 2020 Jul 15;39(17):e104238. doi: 10.15252/embj.2019104238 (PMC7459425; doi:10.15252/embj.2019104238)
Supplement: Supplementary file 1 — Appendix [file EMBJ-39-e104238-s001.pdf]

APPENDIX Montesinos et al.

Table of contents:

Appendix Fig. S1 | CMT patterns in the cytokinin receptor mutants.

Appendix Fig. S2 | CMT arrangements in mutants lacking the cytokinin receptors AHK2 and AHK3.

Appendix Fig. S3 | Cytokinin prevents CMT depolymerisation in the *ahk2-2* and *ahk3-3* cytokinin receptor mutants.

Appendix Fig. S4 | Cytokinin prevents CMT depolymerization in *ahk2-2* and *ahk3-3* cytokinin receptor mutants.

Appendix Fig. S5 | Cytokinin interferes with the auxin-mediated regulation of CMTs.

Appendix Figure S1

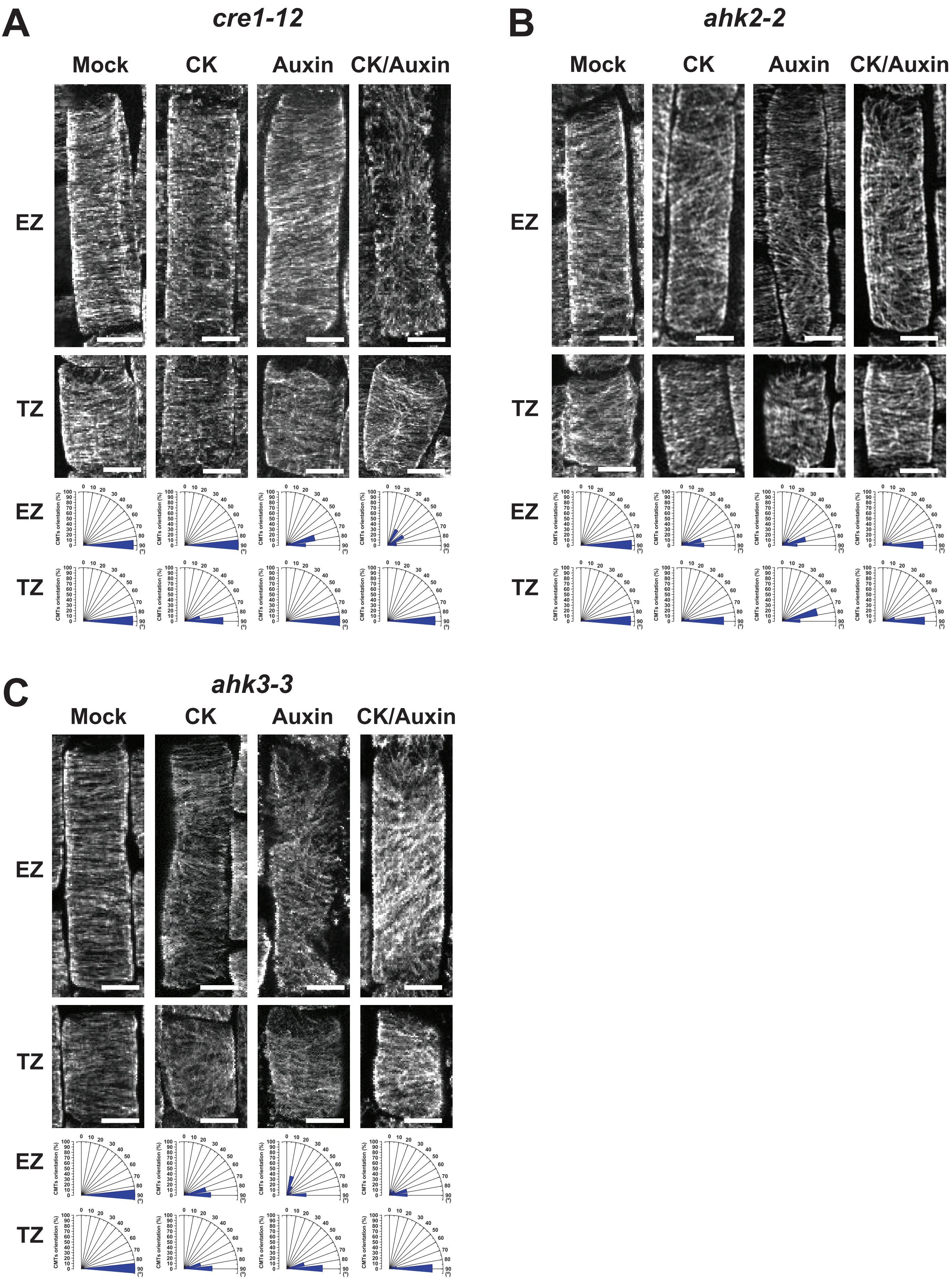

Appendix Fig. S1 | CMT patterns in the cytokinin receptor mutants. A-C. Monitoring of CMTs visualized by immunostaining of α-tubulin and histograms of CMT orientation distributions in epidermal cells of the transition (TZ) and the elongation (EZ) zone of *cre1-12* (**A**), *ahk2-2* (**B**) and *ahk3-3* (**C**) roots. CMTs were visualized in roots treated for 60 min with mock (DMSO), CK (10 µm BAP), auxin (0.1 µM NAA), or CK and auxin (10 µM BAP and 0.1 µM NAA provided after a 60-min pretreatment with 10 µM BAP). n = 10-20 cells per root growth zone in 5-to 8 roots per condition. Scale bar 10 µm.

Appendix Figure S2

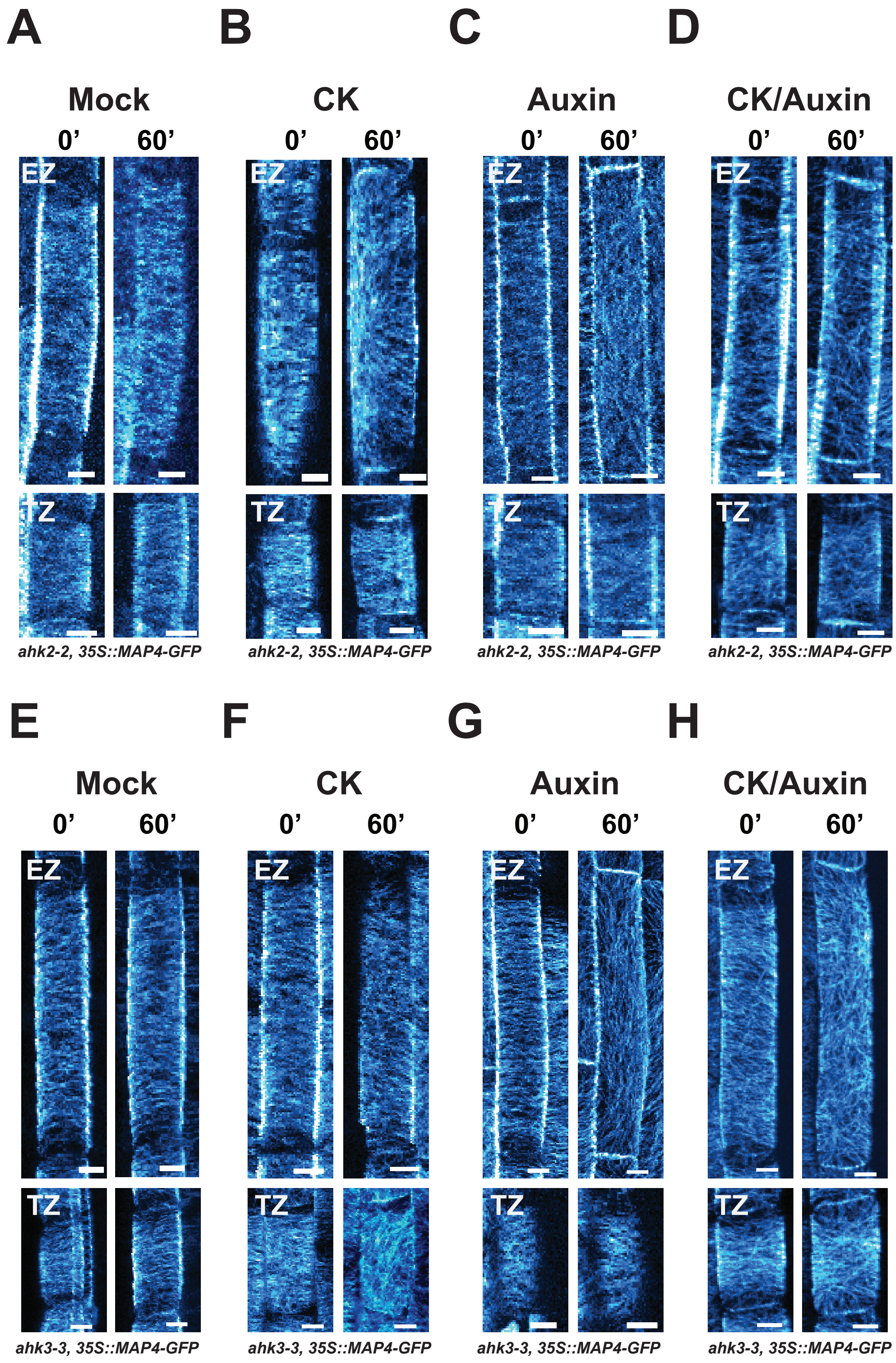

Appendix Fig. S2 | CMT arrangements in mutants lacking the cytokinin receptors AHK2 and AHK3. A-H. Monitoring of CMTs visualized by MAP4-GFP in epidermal cells of the transition (TZ) and elongation (EZ) zone of *ahk2-2* and *ahk3-3* roots. CMTs were visualized in roots treated for 60 min with mock (DMSO) (**A**, **E**), cytokinin (CK, 10 µm BAP) (**B**, **F**), auxin (0.1 µM NAA) (**C**, **G**) or CK and auxin (10 µM BAP and 0.1 µM NAA provided after a 60-min pretreatment with 10 µM BAP) (**D**, **H**). Scale bar 10 µm.

Appendix Figure S3

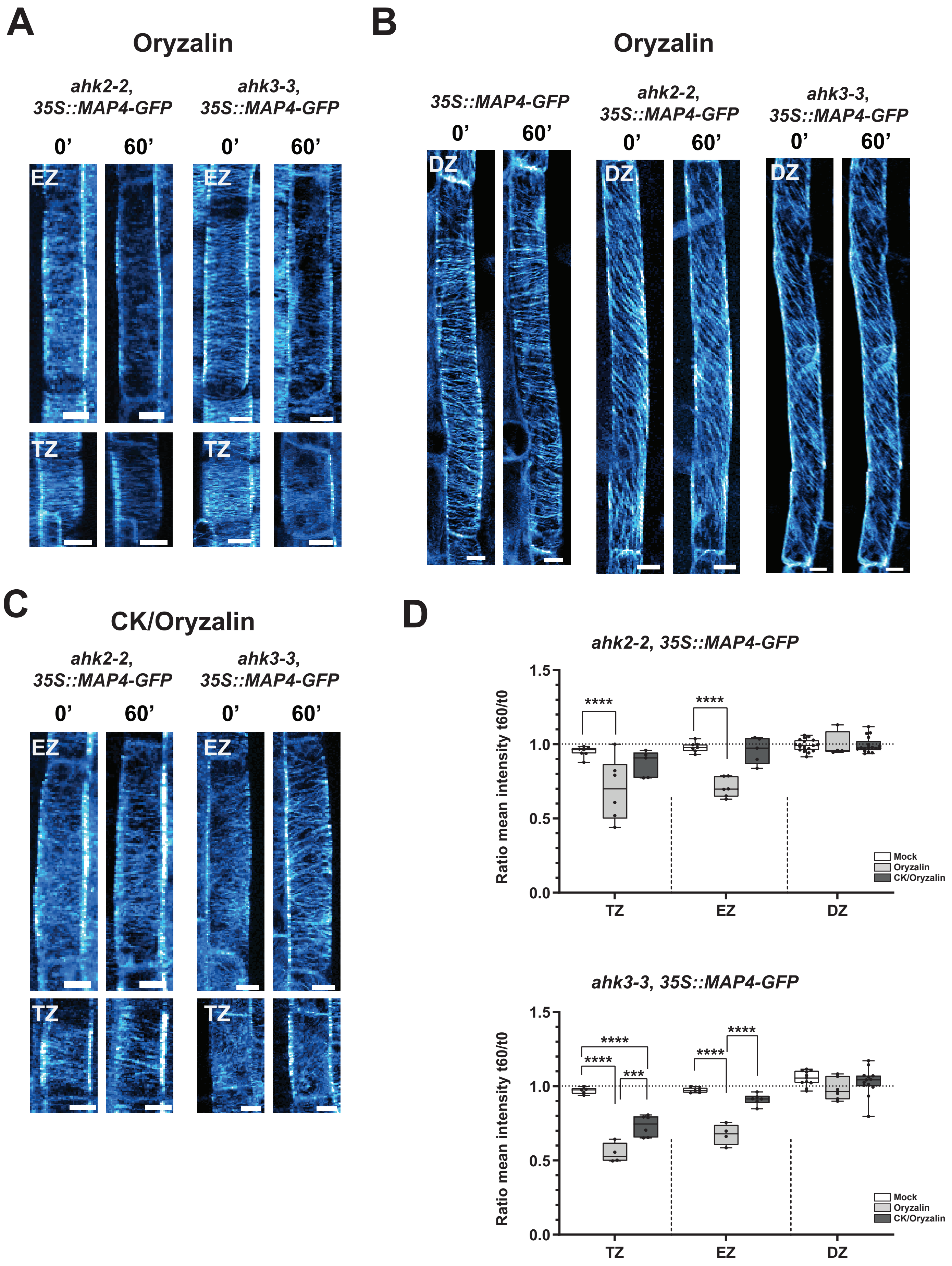

Appendix Fig. S3 | Cytokinin prevents CMT depolymerisation in the *ahk2-2* and *ahk3-3* cytokinin receptor mutants. A-C. CMTs visualized with MAP4-GFP in root epidermal cells of the transition (TZ) and the elongation (EZ) (**A**, **C**) and the differentiation (DZ) (**B**) zone treated with 1 µM oryzalin (**A, B**), and cytokinin (CK, 10 µM BAP) plus oryzalin (1 µM) (**C**). For the double CK and oryzalin treatment, roots were pretreated for 60 min with CK and then transferred to medium supplemented with both compounds. Scale bar 10 µm. **D.** Quantification of the MAP4-GFP reporter signal in *ahk2-2* and *ahk3-3* root epidermal cells of the TZ, the EZ and the DZ treated with mock (DMSO; white box), oryzalin (1 µM; light grey box) and CK and oryzalin (10 µM BAP and 1 µM oryzalin; dark grey box). For the double treatments, roots were pretreated for 60 min with 10 µM BAP prior to transfer to medium supplemented with both compounds. Boxplots represent ratio between mean fluorescence intensity (arbitrary units) measured in epidermal cells at 60 and 0 min. Ratio close to 1 (segmented line) corresponds to an unchanged MAP-GFP signal for 60 min. (*** $P < 0.001$ and **** $P < 0.0001$ by Student's *t*-test, n= 3-10 cells per root growth zone per with 5-8 roots per condition in 3 independent replicates).

Appendix Figure S4

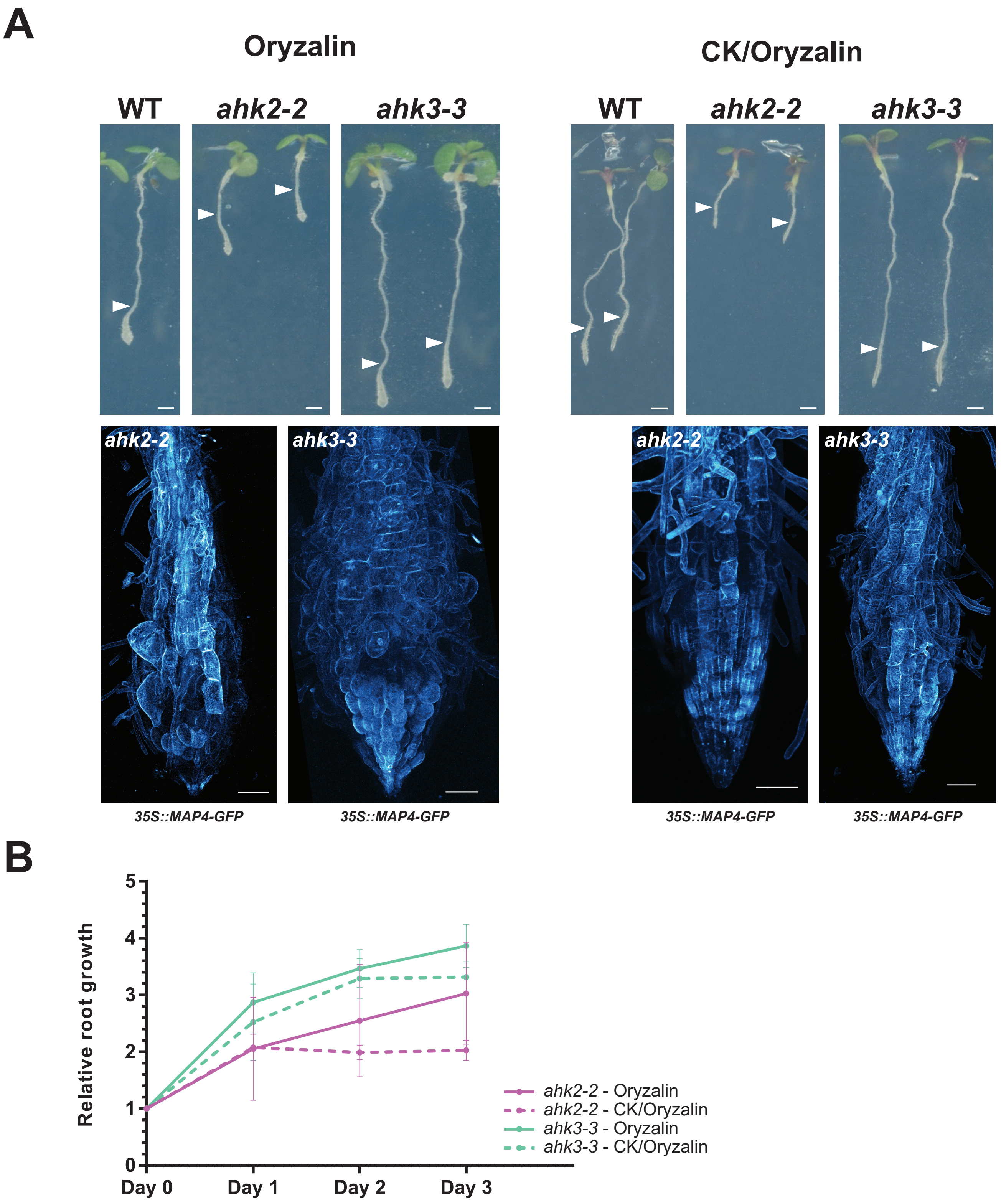

Appendix Fig. S4 | Cytokinin prevents CMT depolymerization in *ahk2-2* and *ahk3-3* cytokinin receptor mutants. A. Representative images of *ahk2-2,35S::MAP4-GFP* and *ahk3-3,35S::MAP4-GFP* seedlings (top) and root tips (bottom) grown for 5 days on mock (Murashige and Skoog) and then transferred to medium supplemented with 1 µM oryzalin, or cytokinin (CK) and oryzalin (10 µM BAP and 1 µM oryzalin) for 3 days. For the double CK and oryzalin treatment, seedlings were pretreated with 10 µM BAP for 60 min prior to transfer to medium supplemented with both compounds. White arrowheads indicate root length at the day of transfer. Confocal images of root tips (bottom) recorded 3 days after transfer. Root tips and CMTs, visualized by the MAP4-GFP reporter, were less affected by oryzalin when pretreated with cytokinin than those treated with oryzalin only in both *ahk2-2* and *ahk3-3*. Scale bar 50 µm. **B.** Relative root growth of *ahk2-2,35S::MAP4-GFP* (pink lines) and *ahk3-3,35S::MAP4-GFP* (green lines) seedlings grown under the conditions described for (**A**) monitored for 3 days. Day 0, day of transfer. Mean ± s.d.; n = 10 roots.

Appendix Figure S5

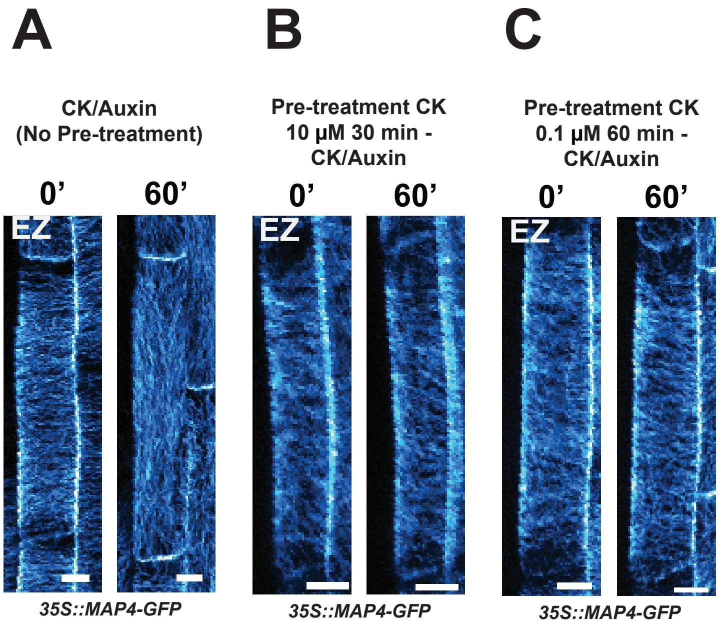

Appendix Fig. S5 | Cytokinin interferes with the auxin-mediated regulation of CMTs. A-C. Monitoring of CMTs visualized by the MAP4-GFP reporter in epidermal cells of the elongation zone (EZ) of wild-type roots treated with cytokinin (CK, 10 µM BAP) and auxin (0.1 µM NAA) (**A**, **B**) without (**A**) or pretreated for 30 min with CK (**B**); CK (0.1 µM BAP) and auxin (0.1 µM NAA) applied after a 60-min pretreatment with CK (**C**).
