# Supplementary material for: Phytohormone cytokinin guides microtubule dynamics during cell progression from proliferative to differentiated stage
Source: EMBO J. 2020 Jul 15;39(17):e104238. doi: 10.15252/embj.2019104238 (PMC7459425; doi:10.15252/embj.2019104238)
Supplement: Supplementary file 2 — Expanded View Figures PDF [file EMBJ-39-e104238-s002.pdf]

Expanded View Figures

Figure EV1. Monitoring of cortical microtubules (CMTs) in root epidermal cells.

A Top surface view of an *Arabidopsis thaliana* root. Cell walls were stained with propidium iodide. Trichoblast root epidermal cells are marked in purple. Scale bar 25 μm.

B Immunostaining of α-tubulin in epidermal cells of the transition zone (TZ) and the elongation zone (EZ) of wild-type roots after 60 min of treatment with mock (DMSO), cytokinin (CK, 10 μM BAP), auxin (0.1 μM NAA), or CK and auxin. For the double CK and auxin treatment, roots were pretreated for 60 min with cytokinin and then transferred to medium supplemented with both compounds. Histograms present the CMT orientation distributions (%) in epidermal cells of the TZ and the EZ treated as indicated. n = 10–24 cells per growth zone in five–eight roots per condition were analyzed. Scale bar 10 μm.

C–G EB1b-GFP plus-end trajectories tinted with a color code according to the speed of their growth rates. The EB1b-GFP plus-end trajectories were monitored for 5 min in epidermal root cells and were analyzed by TrackMate plug-in (Fiji) at the elongation zone (EZ) (C, E–G) and at the differentiation zone (DZ) (D) after 60 min of treatment in mock (DMSO) (C, D), CK (10 μM BAP) (E), auxin (0.1 μM NAA) (F), or CK and auxin (10 μM BAP and 0.1 μM NAA) (G). For the double CK and auxin treatment, roots were pretreated for 60 min with CK prior to transfer to medium supplemented with both compounds. Scale bar 10 μm.

H Monitoring of CMTs in epidermal cells of the EZ using *35S::mCherry-TUA5* marker. Roots were incubated for 1 h in mock (DMSO) or CK (BAP 10 μM) supplemented medium. Five minutes of time-lapse videos and 20 μm segment of the cell (yellow dashed lines) were used to perform Kymograph analysis (upper panels), which were quantified by KymoButler software (lower panels with single trajectories included in the quantification are colored and numbered). Average track velocities (μm/min) are represented by boxplots. The center lines show medians; box limits indicate the 25th and 75th percentiles as determined by the GraphPad software; whiskers span minimum to maximum values; and individual data points are represented by dots. ****$P < 0.0001$ by Student's t-test. n = 3 biological replicates per condition, total number of trajectories analyzed n = 127 and n = 118 for mock- and CK-treated roots, respectively.

I, J Analysis of CMTs (I) and root growth (J) recovery after CK treatment. Roots of 5-day-old seedlings were incubated in mock (DMSO; upper panel) or CK (BAP 10 μM; lower panel) containing medium for 1 h and transferred to mock medium (I, J). Time-lapse images capture CMTs visualized by MAP4-GFP in epidermal root cells at the EZ at 0, 1, 2, 2.5, 3, and 5 h after transfer. Schemes of CMT distribution and cell length at every time next to the images. Scale bar 10 μm (I). Relative root growth (mm) measured during 555 min. Seedlings incubated in mock and transfer to mock medium (black line), pretreated with CK and transferred to mock medium (gray line), pretreated with CK and transferred to CK containing medium (blue line). Mean ± s.d.; n = 10–18 roots per condition.

© 2020 The Authors

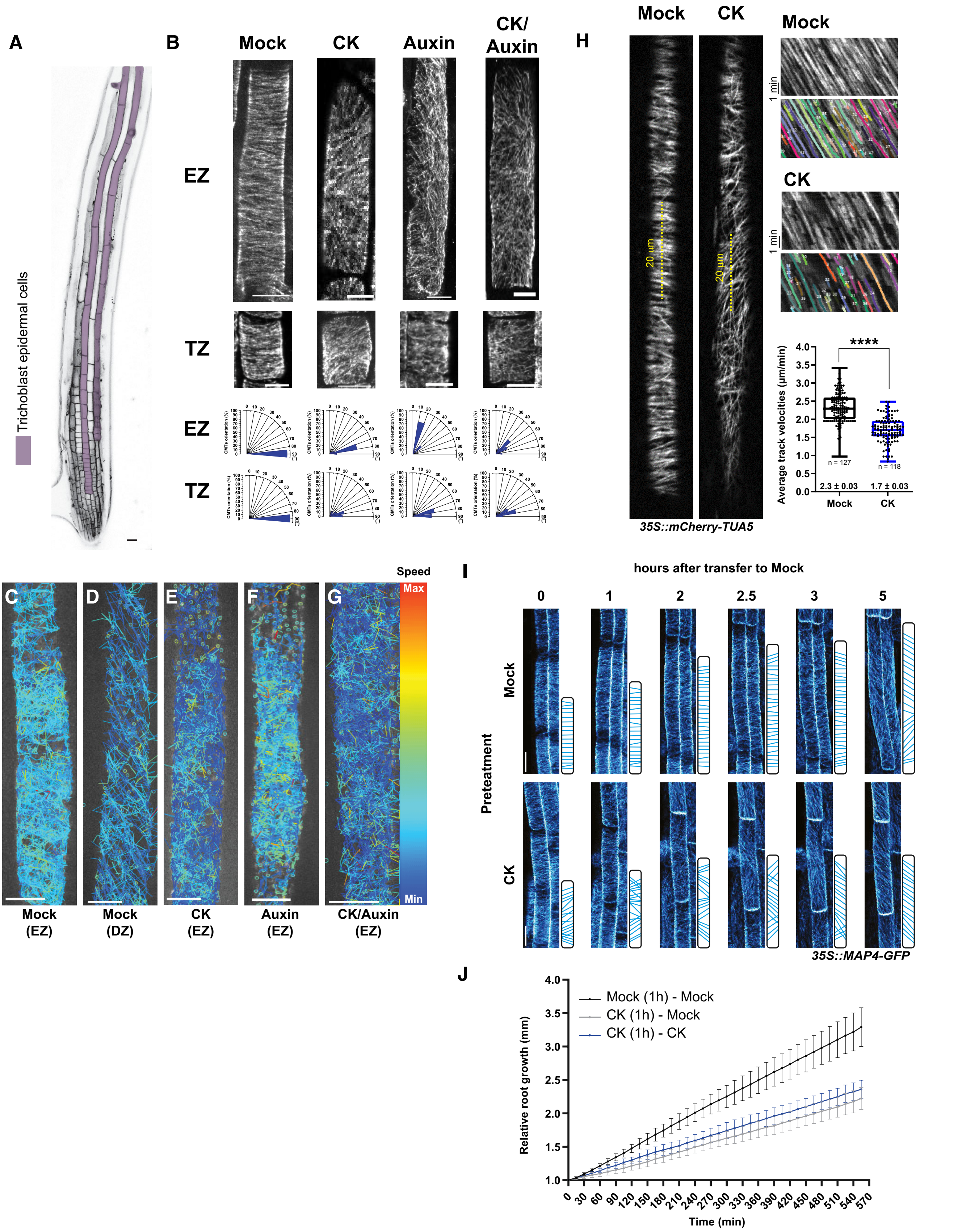

Figure EV1.

 © 2020 The Authors

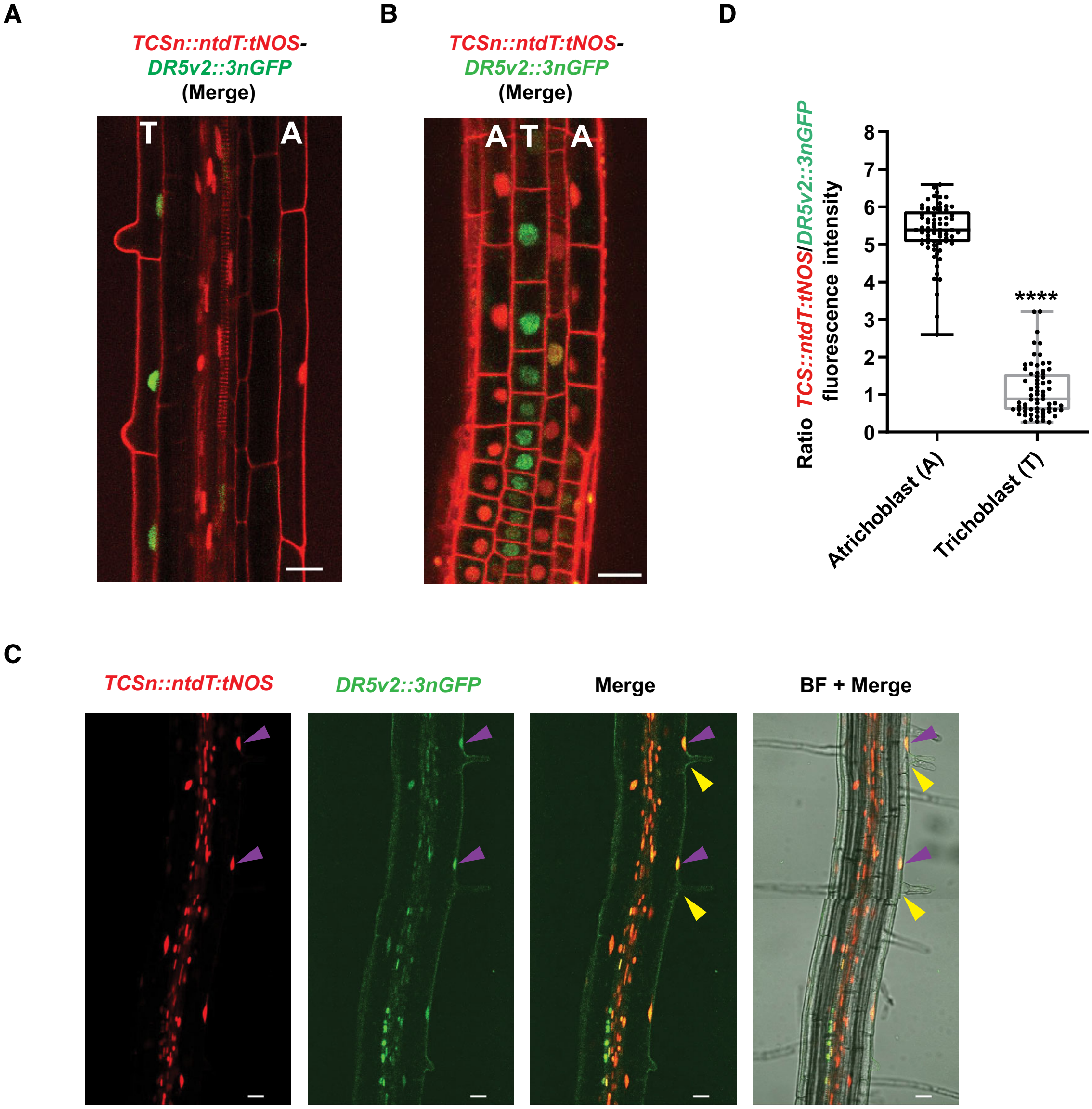

Figure EV2. Monitoring of cytokinin and auxin responses in trichoblast and atrichoblast cells.

A–C Cytokinin (*TCSn::dndT:tNOS*, red) and auxin (*DR5v2::3nGFP*, green) reporter expressions in root atrichoblast (A) and trichoblast (T) in epidermal cells at the beginning of the differentiation zone (DZ) (A), a top surface view of the root transition and elongation zones (B), and in the upper DZ with formed root hairs (purple arrowheads point at the reporter signal; yellow arrowheads indicate root hairs) (C). BF, bright-field picture. Scale bar 25 μm.

D Quantification of the CK and auxin signaling in root atrichoblast (A) and trichoblast (T) epidermal cells at the DZ. Boxplots represent ratio *TCS::ntdT:tNOS* (red)/*DR5v2::3nGFP* (green) fluorescence. The center lines show the medians, and the box limits indicate the 25th and 75th percentiles; whiskers span the minimal to maximal values, and individual data points are represented by dots (****P < 0.0001 by Student's t-test, n = 71 A cells and 67 T cells).

Figure EV3. Cytokinin interferes with depolymerization of CMTs by oryzalin.

A, B Representative images of seedlings (A) and root tips (B) of wild type (Col-0, *35S::MAP4-GFP*), *cre1-12* (*35S::MAP4-GFP*), and *arr1-3* grown for 5 days on mock (Murashige and Skoog) and then transferred to medium supplemented with 1 μM oryzalin or cytokinin (CK, 10 μM BAP) with oryzalin (1 μM oryzalin) for 3 days. For the double CK and oryzalin treatment, seedlings were pretreated with 10 μM BAP for 60 min prior to transfer to medium supplemented with both compounds. White arrowheads indicate root length at day of transfer. Scale bar 1 mm (A). Confocal images of root tips recorded 3 days after transfer. CMTs, visualized by MAP4-GFP reporter, are less affected by oryzalin in wild-type roots pretreated with CK (white arrows) than those treated with oryzalin only. CK pretreatment did not reduce the sensitivity of CMTs to oryzalin in *cre1-12*. Scale bar 50 μm (B).

C Relative root growth of wild type (Col-0, *35S::MAP4-GFP*) (gray lines), *cre1-12 (35S::MAP4-GFP*) (blue lines), and *arr1-3* (orange lines) seedlings grown as described for (A,B) and monitored over 3 days. Day 0, day of transfer. Mean ± s.d.; n = 15–18 roots.

D Visualization of CMTs by immunostaining of α-tubulin in epidermal cells of the transition zone (TZ) and the elongation zone (EZ) of *arr1-3* roots after 60-min treatment with mock (DMSO), CK (10 μM BAP), auxin (0.1 μM NAA), or CK and auxin. For the double CK and auxin treatment, roots were pretreated for 60 min with cytokinin and then transferred to medium supplemented with both compounds. Scale bar 10 μm.

E Histograms present the CMT orientation distributions (%) in *arr1-3* epidermal cells of the TZ and the EZ treated as described in (D). n = 12–20 cells per growth zone in 5–8 roots per condition were analyzed.

 © 2020 The Authors

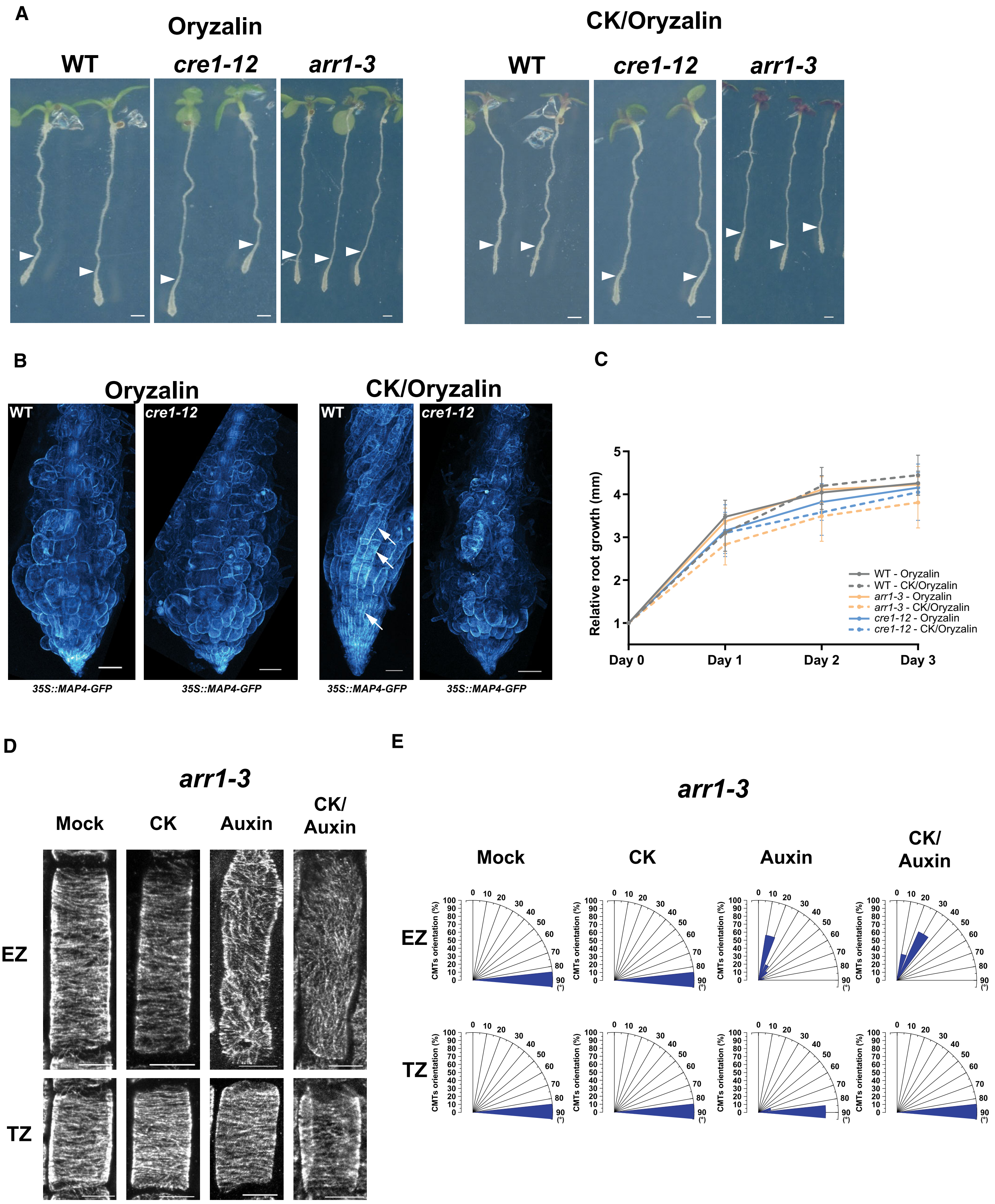
A
Oryzalin
CK/Oryzalin
WT
cre1-12
arr1-3
B
Oryzalin
CK/Oryzalin
WT
cre1-12
35S::MAP4-GFP
C
Relative root growth (mm)
Day 0
Day 1
Day 2
Day 3
WT - Oryzalin
WT - CK/Oryzalin
arr1-3 - Oryzalin
arr1-3 - CK/Oryzalin
cre1-12 - Oryzalin
cre1-12 - CK/Oryzalin
D
arr1-3
Mock
CK
Auxin
CK/ Auxin
EZ
TZ
E
arr1-3
Mock
CK
Auxin
CK/ Auxin
EZ
TZ
CMTs orientation (%)
(°)

Figure EV3.

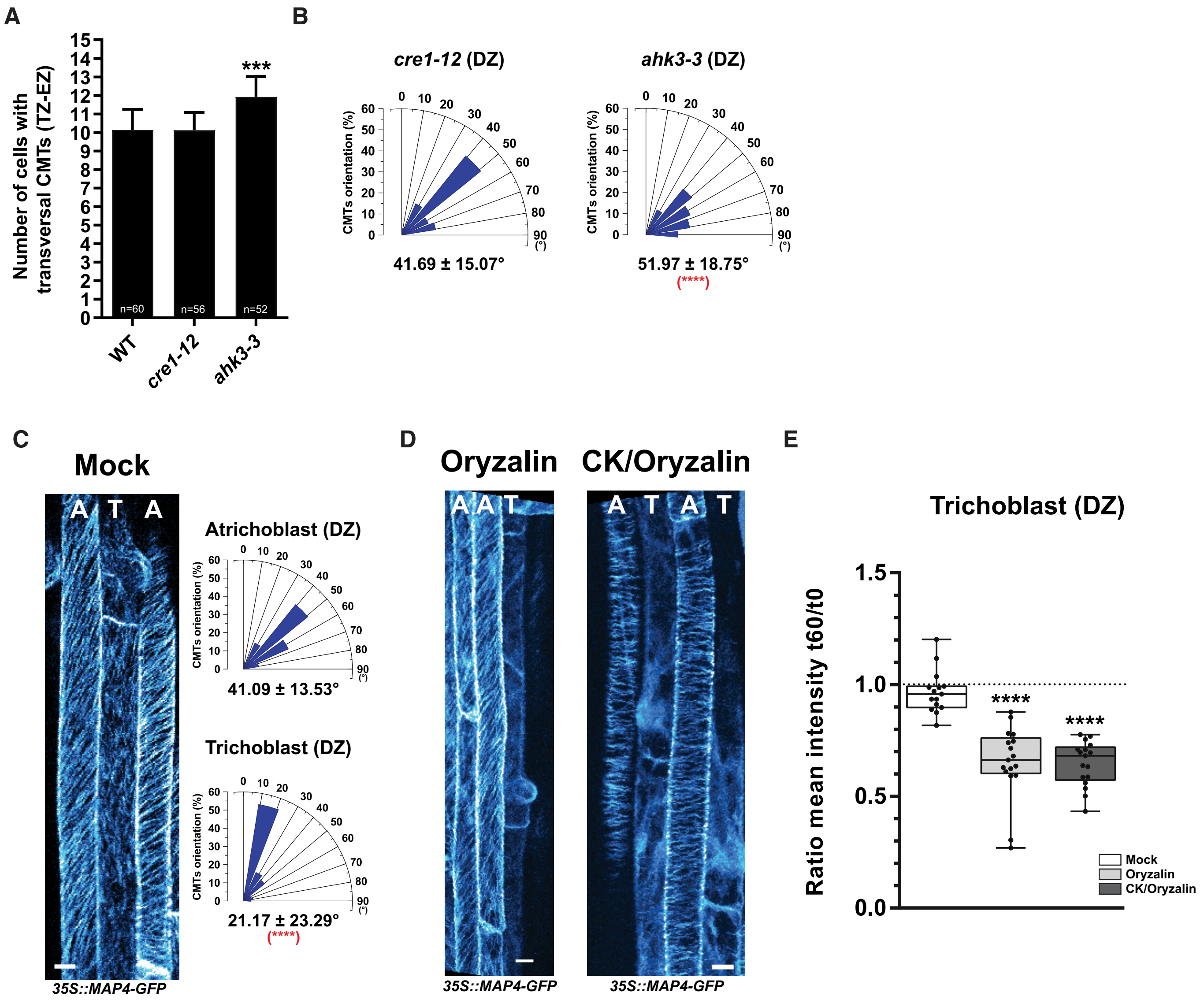

Figure EV4. Cytokinin through cytokinin receptors fine-tunes CMT arrangements in root epidermal cells.

A Number of epidermal cells with transversal disposition of CMTs along the longitudinal root growth axis quantified in 5-day-old wild type (WT), *cre1-12*, and *ahk3-3* (CMTs visualized using MAP4-GFP). Mean ± s.d., (****P* < 0.001 by Student's *t*-test, *n* = 60, 56, and 52 roots of WT, *cre1-12*, and *ahk3-3*, respectively, in three independent replicates).

B Histograms of the CMT orientation distributions in root epidermal cells of the DZ in *cre1-12* and *ahk3*-3. Orientation of CMTs analyzed in atrichoblasts. Mean ± s.d., (*****P* < 0.0001 by Student's *t*-test, compared to atrichoblast at the DZ in WT, (C); *n* = 116 and 100 cells for *cre1-12* and *ahk3-3*, respectively, in four independent replicates).

C, D CMTs visualized with MAP4-GFP in root epidermal cells of the differentiation zone (DZ) after a 60-min treatment with mock (DMSO) (C), or with oryzalin (1 μM), or cytokinin (CK, 10 μM BAP) plus oryzalin (1 μM) applied after a 60-min pretreatment with 10 μM BAP) (D). Histograms of the CMT orientation distributions in trichoblast (T) and atrichoblast (A) cells at the DZ. Mean ± s.d. (*****P* < 0.0001 by Student's *t*-test, compared to atrichoblasts; *n* = 70 atrichoblasts and *n* = 93 trichoblasts) (C). Scale bar 10 μm.

E Quantification of the MAP4-GFP reporter signal in wild-type root epidermal trichoblast cells (T) of the differentiation zone (DZ) treated with mock (DMSO, white box), oryzalin (1 μM, light gray box), and CK (10 μM BAP) plus oryzalin (1 μM) (dark gray box). For double treatments, roots pretreated for 60 min with CK prior to transfer to medium supplemented with both compounds. Boxplots represent ratio between mean fluorescence intensity (arbitrary units) measured in epidermal cells at 60 and 0 min. The center lines show the medians, and the box limits indicate the 25th and 75th percentiles; whiskers span the minimal to maximal values, and individual data points are represented by dots. Ratio close to 1 (segmented line) corresponds to the unchanged MAP-GFP signal for 60 min (*****P* < 0.0001 by Student's *t*-test compared to mock, *n* = 15–17 cells per root growth zone with 4–6 roots per condition in three independent replicates).

© 2020 The Authors

Figure EV5. Cytokinin derivatives regulate MT dynamics in leukocytes.

A Averages of MT plus-end growth rates (μm/min) of untreated leukocytes expressing EB3-mCherry, n = 5 cells.

B Relative MT plus-end growth speeds (μm/min) of leukocytes expressing EB3-mCherry after mock (DMSO), cytokinin (CK, 10 μM BAP or 10 μM *trans*-zeatin), and auxin (0.1 μM NAA) treatment (as described for Fig 6A) compared to untreated cells. Mean ± s.d., ns, non-significant, *P < 0.05, **P < 0.01, by Student's t-test (n = 4–6 cells per condition in three independent replicates).

C Histograms of MT plus-end growth rate (μm/min) distribution frequencies in leukocytes expressing EB3-mCherry before (gray) and after (purple) treatment with mock (DMSO), CK (10 μM BAP or 10 μM trans-zeatin), and auxin (0.1 μM NAA). MT plus-end growth rates were monitored for 5 min before and for 5 min after each treatment (as described for Fig 5A).

D Time projection over 30 s of individual leukocytes expressing the plus-end protein marker EB3-mCherry, before and after treatment with mock (DMSO) or CK (10 μM BAP). Pink arrowheads indicate similar MT trajectories before and after mock (DMSO) treatment. Yellow arrowheads show the shorter MT trajectories after CK (10 μM BAP) treatment than those before the CK treatment. Scale bar 25 μm.

© 2020 The Authors

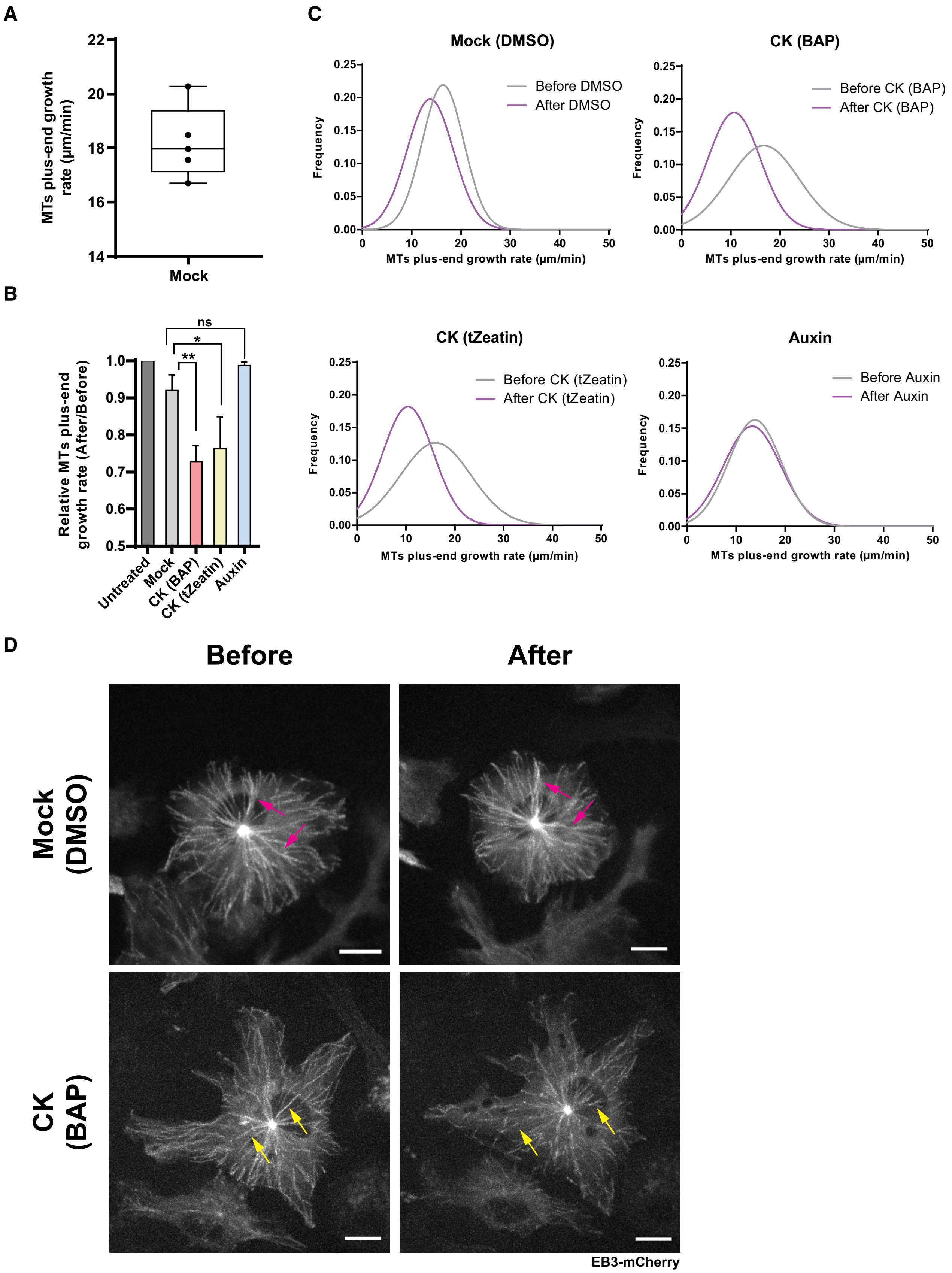

Figure EV5.

© 2020 The Authors
