# Supplementary material for: Phytohormone cytokinin guides microtubule dynamics during cell progression from proliferative to differentiated stage
Source: EMBO J. 2020 Jul 15;39(17):e104238. doi: 10.15252/embj.2019104238 (PMC7459425; doi:10.15252/embj.2019104238)
Supplement: Supplementary file 3 — Table EV1 [file EMBJ-39-e104238-s003.pdf]

Table EV1

	Microtubules orientation (0-90 degrees)				
		Transition zone		Elongation zone	
		0 min	60 min	0 min	60 min
Mock (DMSO)	**WT**	83.42 ± 1.1	82.99 ± 1.3 [a]	84.46 ± 0.9	82.68 ± 1.1 [a]
	cre1-12	79.83 ± 1.4	84.85 ± 1.4 [a]	84.61 ± 1.3	81.77 ± 1.6 [a]
	ahk2-2	74.56 ± 6.1	77.78 ± 3.0 [a]	78.76 ± 2.4	80.72 ± 1.8 [a]
	ahk3-3	79.68 ± 2.3	81.25 ± 1.3 [a]	84.65 ± 1.2	81.90 ± 1.5 [a]
CK (BAP 10 µM)	**WT**	81.03 ± 1.3	64.08 ± 4.6 [a]/**	82.78 ± 0.9	56.93 ± 5.4 [a]/****
	cre1-12	82.57 ± 1.8	77.80 ± 2.3 [b]	82.87 ± 1.5	82.15 ± 1.8 [bc]
	ahk2-2	83.59 ± 1.4	78.58 ± 1.6 [b]/*	80.90 ± 2.7	71.73 ± 6.2 [ac]/*
	ahk3-3	81.19 ± 1.4	62.56 ± 3.7 [a]/***	82.01 ± 1.7	62.15 ± 4.7 [a]/***
Auxin (NAA 0.1 µM)	**WT**	80.39 ± 2.2	61.71 ± 7.8 [a]/*	80.25 ± 1.6	7.50 ± 1.8 [a]/****
	cre1-12	77.52 ± 3.1	70.28 ± 3.01 [a]	82.54 ± 1.0	42.79 ± 5.8 [bc]/****
	ahk2-2	80.15 ± 2.1	63.79 ± 7.8 [a]/*	81.96 ± 2.0	20.32 ± 5.3 [a]/****
	ahk3-3	83.28 ± 2.0	63.45 ± 3.8 [a]/***	85.32 ± 1.0	31.72 ± 8.2 [ac]/****
CK/Auxin (Pre-treatment BAP 10 µM 1h - NAA 0.1 µM + BAP 10 µM)	**WT**	71.65 ± 2.5	69.06 ± 7.1 [ab]	74.73 ± 2.9	67.85 ± 5.2 [a]
	cre1-12	73.56 ± 4.7	77.42 ± 1.9 [a]	71.20 ± 4.0	34.22 ± 5.2 [b]/****
	ahk2-2	68.87 ± 4.5	47.92 ± 6.8 [b]	55.52 ± 6.6	32.53 ± 5.7 [b]/*
	ahk3-3	75.49 ± 2.3	70.30 ± 6.2 [ab]	42.96 ± 7.3	21.21 ± 6.4 [b]/*

Referred to time 0 (time 60min vs time 0 min)

Referred to same condition (mock, CK, Auxin or CK/Auxin) and same zone (TZ or EZ)

Anova $P < 0.05$

Table EV1 | CMT orientation in root epidermal cells of the wild type and the cytokinin receptor mutants. Average angle of the CMT orientations was measured in the wild type (WT) and the *cre1-12*, *ahk2-2* and *ahk3-3* mutants 0 and 60 min after the respective treatments as indicated. Mean ± s.d.. Statistical significance evaluated by Student's *t*-test (* $P < 0.05$, ** $P < 0.01$, *** $P < 0.001$ or **** $P < 0.0001$; referred to time 0) and two-way ANOVA (a-c = $P < 0.05$; same growth zone of wild-type, *cre1-12*, *ahk2-2*, *ahk3-3* mutants in same treatment conditions compared). n = more than 10 cells per growth zone in more than 5 roots per conditions in 3 independent replicates.
