# Supplementary material for: Phytohormone cytokinin guides microtubule dynamics during cell progression from proliferative to differentiated stage
Source: EMBO J. 2020 Jul 15;39(17):e104238. doi: 10.15252/embj.2019104238 (PMC7459425; doi:10.15252/embj.2019104238)
Supplement: Supplementary file 4 — Movie EV1 [file EMBJ-39-e104238-s004.zip › Movie EV1.rtf]

Movie EV1 | CMT orientation changes from transversal to oblique at the beginning of the DZ. Arabidopsis thaliana roots expressing the CMT marker MAP4-GFP monitored for 120 min (1 picture/10 min) by means of a vertical confocal microscope. White arrowheads indicate epidermal root cells at the end of the EZ entering the DZ. Scale bar 100 µm.
