# Supplementary material for: Phytohormone cytokinin guides microtubule dynamics during cell progression from proliferative to differentiated stage
Source: EMBO J. 2020 Jul 15;39(17):e104238. doi: 10.15252/embj.2019104238 (PMC7459425; doi:10.15252/embj.2019104238)
Supplement: Supplementary file 5 — Movie EV2 [file EMBJ-39-e104238-s005.zip › Movie EV2.rtf]

Movie EV2-6 | Monitoring of CMT plus-end growths. CMT plus-end growths were visualized with the EB1b-GFP marker in epidermal root cells at the elongation (EZ) (2, 4, 5, 6) and at the differentiation (DZ) (3) zone in mock (DMSO) conditions (2, 3) or after 60-min treatment with cytokinin (CK, 10 µM BAP) (4), auxin (0.1 µM NAA) (5) or CK plus auxin (10 µM BAP and 0.1 µM NAA applied after a 60-min pretreatment with 10 µM BAP) (6). Individual trajectories of CMT plus-end growths are recorded and visualized with EB1b-GFP tracked for 5 min by TrackMate plug-in (Fiji). Note the transversal (2), oblique (3, 4 and 6) and longitudinal (5) direction of CMT plus-end growths. Scale bar 10 µm.
