# Supplementary material for: Phytohormone cytokinin guides microtubule dynamics during cell progression from proliferative to differentiated stage
Source: EMBO J. 2020 Jul 15;39(17):e104238. doi: 10.15252/embj.2019104238 (PMC7459425; doi:10.15252/embj.2019104238)
Supplement: Supplementary file 10 — Movie EV7 [file EMBJ-39-e104238-s010.zip › Movie EV7.rtf]

Movie EV7 and EV8 | Monitoring of CMTs orientation after transient cytokinin treatment. Arabidopsis thaliana epidermal roots expressing the CMT marker MAP4-GFP monitored for 360 min (1 picture/5 min) by means of a vertical confocal microscope in mock medium after 1 h pretreatment with either mock (DMSO; Movie EV7) or cytokinin (CK, BAP 10 µM; Movie EV8). White arrowheads indicate epidermal root cells at the EZ. Scale bar 50 µm.
