# Supplementary material for: Phytohormone cytokinin guides microtubule dynamics during cell progression from proliferative to differentiated stage
Source: EMBO J. 2020 Jul 15;39(17):e104238. doi: 10.15252/embj.2019104238 (PMC7459425; doi:10.15252/embj.2019104238)
Supplement: Supplementary file 12 — Movie EV9 [file EMBJ-39-e104238-s012.zip › Movie EV9.rtf]

Movie EV9 | Monitoring of CMT plus-end growths in trichoblasts. CMT plus-end growths were visualized with the EB1b-GFP marker in trichoblasts cells in mock conditions. Individual trajectories of CMT plus-end growths are recorded and visualized with EB1b-GFP tracked for 5 min. Scale bar 10 µm.
