# Supplementary material for: Phytohormone cytokinin guides microtubule dynamics during cell progression from proliferative to differentiated stage
Source: EMBO J. 2020 Jul 15;39(17):e104238. doi: 10.15252/embj.2019104238 (PMC7459425; doi:10.15252/embj.2019104238)
Supplement: Supplementary file 13 — Movie EV10 [file EMBJ-39-e104238-s013.zip › Movie EV10.rtf]

Movie EV10 and EV11 | Leukocytes expressing MT plus-end marker EB3-mCherry. Movies recorded for 5 min showing the growth of MT comets before (Movie EV10) and immediately after treatment (Movie EV11) with mock (DMSO; left) and cytokinin (CK, BAP 10 µM; right). Scale bar 25 µm.
